# Double Virus Vector Infection to the Prefrontal Network of the Macaque Brain

**DOI:** 10.1371/journal.pone.0132825

**Published:** 2015-07-20

**Authors:** Mineki Oguchi, Miku Okajima, Shingo Tanaka, Masashi Koizumi, Takefumi Kikusui, Nobutsune Ichihara, Shigeki Kato, Kazuto Kobayashi, Masamichi Sakagami

**Affiliations:** 1 Brain Science Institute, Tamagawa University, Machida, Tokyo, Japan; 2 Graduate School of Arts and Sciences, The University of Tokyo, Meguro, Tokyo, Japan; 3 School of Veterinary Medicine, Azabu University, Sagamihara, Kanagawa, Japan; 4 Department of Molecular Genetics, Institute of Biomedical Sciences, Fukushima Medical University, Fukushima, Fukushima, Japan; University of Pittsburgh School of Medicine, UNITED STATES

## Abstract

To precisely understand how higher cognitive functions are implemented in the prefrontal network of the brain, optogenetic and pharmacogenetic methods to manipulate the signal transmission of a specific neural pathway are required. The application of these methods, however, has been mostly restricted to animals other than the primate, which is the best animal model to investigate higher cognitive functions. In this study, we used a double viral vector infection method in the prefrontal network of the macaque brain. This enabled us to express specific constructs into specific neurons that constitute a target pathway without use of germline genetic manipulation. The double-infection technique utilizes two different virus vectors in two monosynaptically connected areas. One is a vector which can locally infect cell bodies of projection neurons (local vector) and the other can retrogradely infect from axon terminals of the same projection neurons (retrograde vector). The retrograde vector incorporates the sequence which encodes Cre recombinase and the local vector incorporates the “Cre-On” FLEX double-floxed sequence in which a reporter protein (mCherry) was encoded. mCherry thus came to be expressed only in doubly infected projection neurons with these vectors. We applied this method to two macaque monkeys and targeted two different pathways in the prefrontal network: The pathway from the lateral prefrontal cortex to the caudate nucleus and the pathway from the lateral prefrontal cortex to the frontal eye field. As a result, mCherry-positive cells were observed in the lateral prefrontal cortex in all of the four injected hemispheres, indicating that the double virus vector transfection is workable in the prefrontal network of the macaque brain.

## Introduction

Dissecting the function of a specific neural circuit is crucial for understanding how the information processing of the brain works. To achieve this goal, pathway-specific and neuron type- specific intervention is necessary. Recently, a number of studies have made remarkable advances in optogenetic and pharmacogenetic approaches used to regulate specific neural pathways [1, 2, 3]. Some studies have utilize transgenic animals (which express site-specific recombinase in a specific type of neuron) and inject virus vector which includes recombinase-dependent expressing sequence into connected brain areas. The application of this technique, however, has been restricted to animals other than primates. The double virus vector infection method, in which two different types of virus vectors are injected into two monosynaptically connected areas, is a prospective method to regulate a specific neural pathway of primates. A previous study applied this method to the spinal cord of the macaque monkey and observed impairment of the hand movement dexterity under pharmacogenetic blockade of the indirect pathway [4]. Two other studies applied this method to the rodent’s brain and reported behavioral effects [5, 6]. The next step is to apply this technique to neural pathways in the cerebrum of the macaque monkey. Thus far, however, no one has yet demonstrated that the double-infection technique works in the primate brain.

Here, we introduced the double virus vector infection into the macaque brain. Macaque monkeys have a bunch of higher cognitive functions in common with humans due to their well-developed frontal lobes. Advancing the double-infection technique for use on such animals has the potential of being able to uncover neural bases of these common cognitive functions. We therefore targeted two pathways in the prefrontal network: the frontostriatal pathway from the lateral prefrontal cortex (LPFC) to the caudate nucleus (Cd) and the frontofrontal pathway from the LPFC to the frontal eye field (FEF). Previous studies have suggested that frontostriatal connections are responsible for important cognitive functions, such as cognitive flexibility, task shifting, and inhibiting impulsive behaviors [7, 8], and that these connections are implicated in neurological and psychiatric disorders, such as autism, schizophrenia, and obsessive-compulsive disorder [9, 10, 11]. Different prefrontal areas one-directionally project to slightly different areas in the striatum [12, 13, 14]. Among these areas, LPFC has a strong connection especially with the dorsal part of Cd. LPFC also has a connection with FEF [15], which is located very close to LPFC. This frontofrontal pathway has been suggested to be involved in inhibitory control of the oculomotor behavior [16, 17].

In this study, the adeno-associated viral vector type 5 (AAV5), which has high efficiency of infection to cell bodies of neurons in its injection site [18, 19], was injected into the LPFC. Two different vectors, AAV9 and Highly efficient Retrograde gene transfer (HiRet) virus vector, which both are highly infectable from axon terminals in the injection site and then retrogradely transport to their cell bodies [20, 21, 22, 23], were injected into Cd/FEF. While AAV9/HiRet encoded Cre-recombinase and enhanced green fluorescent protein (eGFP), AAV5 encoded the “Cre-On” FLEX double-floxed sequence, in which red fluorescent protein (mCherry) was included. This double-infection technique leads to the expression of mCherry only in double-infected LPFC neurons that extend their axon-terminals to the Cd/FEF. The FLEX sequence also included newly-developed engineered G-protein coupled receptor: the Designer Receptors Exclusively Activated by the Designer Drugs (DREADDs), whose activities can be modulated exclusively and reversibly by administering a specific extrinsic ligand: clozapine-N-oxide (CNO) [24, 25]. The activity of neurons that express excitatory type (hM3D_q_) increase, while the activity of neurons that express inhibitory type (hM4D_i_) decrease, when administering CNO. The aim of the inclusion of DREADDs in the AAV5 is to test the feasibility of DREADD expression on the specific prefrontal neurons in the macaque brain for future pharmacogenetic manipulation studies. Although we did not test the behavioral and neuronal effects of CNO administration in this study, the observation of mCherry-positive neurons highly likely indicates DREADD expression in our double infection system. After the sufficient waiting period from extensive double virus vector injection, mCherry-positive cells were observed in the LPFC areas in all injected hemispheres, indicating that double-infection is a workable technique for the prefrontal network of the macaque brain.

## Materials and Methods

### Animals

Two male Japanese monkeys (*Macaca fuscata*) were used in this study (Monkey TA, 8.0 kg, and Monkey TO, 9.7 kg). We implanted a head holder and two recording chambers for each monkey according to the conventional way [26]. Each recording chamber (Monkey TA: 30 mm length, anterior—posterior, and 30 mm width, lateral-medial. Monkey TO: 42 mm length, anterior—posterior, and 30 mm width, lateral-medial) was implanted with its center located near the end of the principal sulcus. No behavioral abnormalities, loss of appetite, loss of weight, or other abnormalities was observed in either of these monkeys during the experiment.

In addition, a male adult C57BL/6 mouse (Crea Japan Inc., Tokyo, Japan) was used for the confirmation of the validity of the FLEX system.

### Ethics Statement

All experimental protocols in this macaque study were approved by the Animal Care and Use Committee (H26-42) and the safety committee for genetic modification research (C26-03) at Tamagawa University and were in accordance with the National Institutes of Health’s *Guide for the Care and Use of Laboratory Animals*. The monkeys were kept in individual primate cages in an air-conditioned room where food and water were available ad libitum. Each cage was equipped with a platform along the middle of the wall which enabled the monkeys to freely move up and down. The body weight and appetite of the monkeys were checked and vegetables and fruits were provided daily. The monkeys were seated in a primate chair during the experiment with their heads fixed. We constantly delivered additional water during extra-cellular recording and supplied diced vegetables and fruits during virus vector injection. The inside of the recording chambers were regularly washed with saline and antibiotic ointment was put on the dura matter to keep the inside clean. They were deeply anesthetized with an overdose of sodium pentobarbital (40 mg/kg, i.v.) when being sacrificed.

The procedure of the mouse experiment was approved by the Ethics Committees of both Azabu University and the University of Tokyo, Japan (#130226). The mouse was housed under a standard 12-h light/dark cycle (lights on from 6 a.m. to 6 p.m.), and the environment was maintained at constant temperature (24 ± 1°C) and humidity (50 ± 5%). Food and water were provided ad libitum.

### Viral construction

AAV5-hSyn-DIO-hM4D_i_-mCherry and AAV9-hSyn-GFP-Cre viral vectors (titer: 5.0×10^13^ copies/ml) were purchased from the Gene Therapy Center Vector Core, University of North Carolina at Chapel Hill (http://www.med.unc.edu/genetherapy/vectorcore). Briefly, hM4D_i_-mCherry and hSyn-GFP-Cre coding sequences were amplified by PCR, and the amplicons and an AAV vector with a human Synapsin 1 promoter (http://www.ncbi.nlm.nih.gov/pmc/articles/PMC3069789/) were digested with NheI and AscI. The digestion products were ligated such that the coding regions for the fusion proteins were in a 3’ to 5’ orientation relative to the promoter. The final vectors were sequence verified and packaged in serotype5 and 9, respectively.

A vector for highly efficient retrograde gene transfer (HiRet) by pseudotyping an HIV-1-based vector (titer: 2.6×10^11^ copies/ml) was prepared as described previously [21, 22] with some modifications. Briefly, the transfer plasmid (pCL20c-MSCV-nls/Cre-2A-eGFP) contained the cDNA encoding Cre recombinase fused to a nuclear localization sequence, 2A peptide and enhanced green fluorescent protein (eGFP) downstream of the murine stem-cell virus promoter (MSCV). The envelope plasmid contained the cDNA encoding fusion glycoprotein type B2 (FuG-B2) [22] under the control of the cytomegalovirus enhancer/chicken *β*-actin promoter [27]. HEK293T cells were transfected with the transfer, envelope, and packaging plasmids by using the calcium-phosphate precipitation method. The vector particles were centrifuged at 6,000 X g for 16–18 hrs and resuspended in phosphate-buffered saline (PBS). The particles were applied to a Sepharose Q FF ion-exchange column (GE Healthcare, Buckinghamshire, UK) eluted, and then concentrated by centrifugation through a Vivaspin filter (Vivascience, Lincoln, UK).

### Double-infection

**Fig 1:**
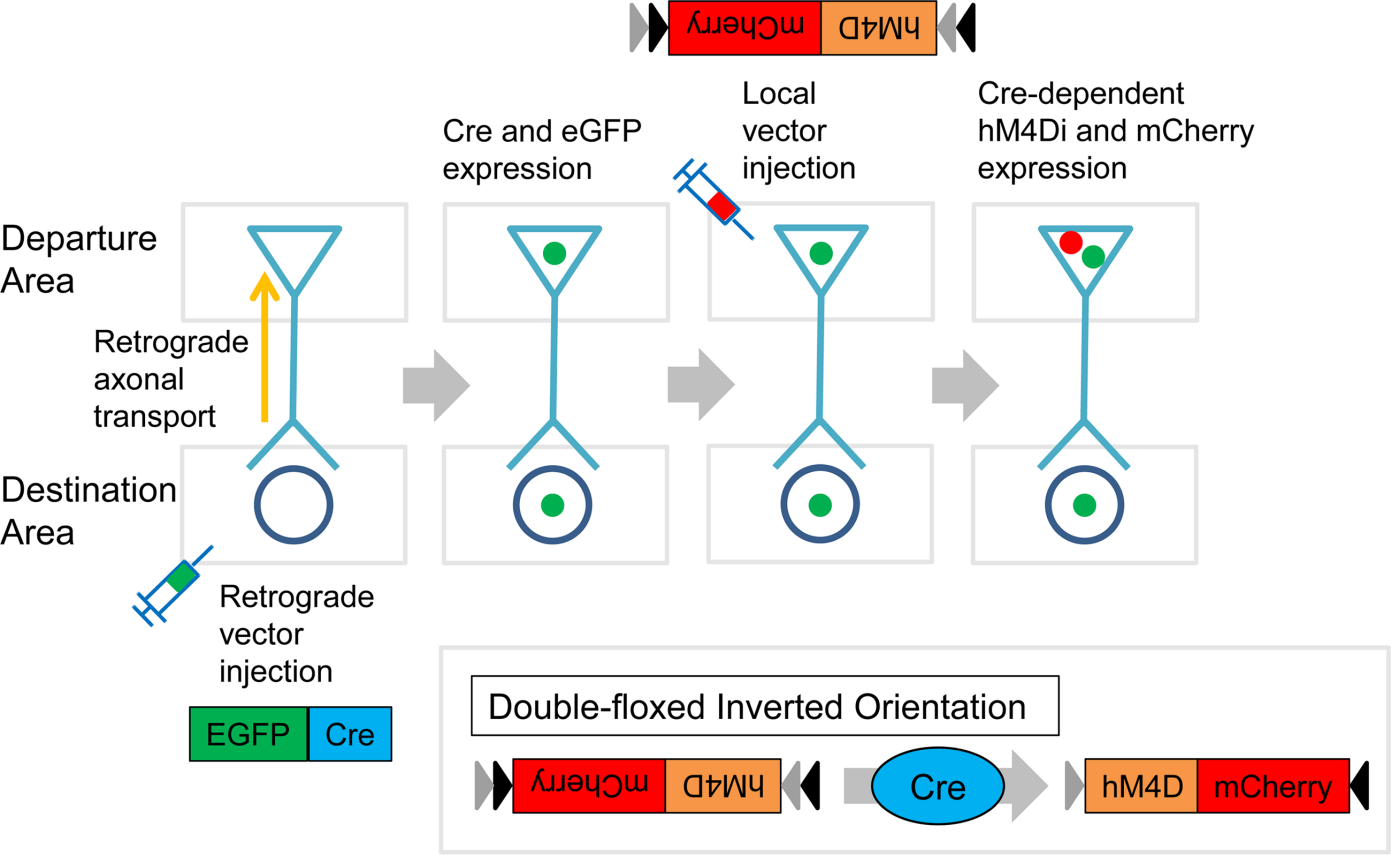
Schematic diagram of the pathway specific gene transfer using the double virus vector infection. The retrograde vector was injected into the destination area: where the axon terminal of the target cell was. The retrograde vector was then transported to the cell body of the target cell through axonal transport. The target cell expressed Cre and eGFP if retrogradely infected by the retrograde vector. The local vector was injected into the departure area: where the soma of the target cell was. Only doubly infected target cell expressed mCherry and possibly hM4D_i_ because of the “Cre-on” system. In our study local vector and retrograde vector were alternately injected over a period of weeks.

The double-infection technique utilizes two different virus vectors in monosynaptically connected areas that constitute some particular projection pathway. One is a vector which can locally infect cell bodies of projection neurons (a “local vector”) and the other is a vector which can retrogradely infect from axon terminals of projection neurons to their cell nuclei (a “retrograde vector”). In this study, the two retrograde vectors that we used (AAV9/HiRet) incorporated the sequence which encodes Cre-recombinase and eGFP, while the local vector that we used (AAV5) incorporated the “Cre-On” FLEX double-floxed sequence in which mCherry and hM4D_i_ were included. The FLEX sequence reverses under Cre-existence condition and the constructs in the FLEX sequence become readable. mCherry and hM4D_i_ thus come to be expressed only in neurons which were doubly infected by both local and retrograde vectors (Fig 1).

**Fig 2:**
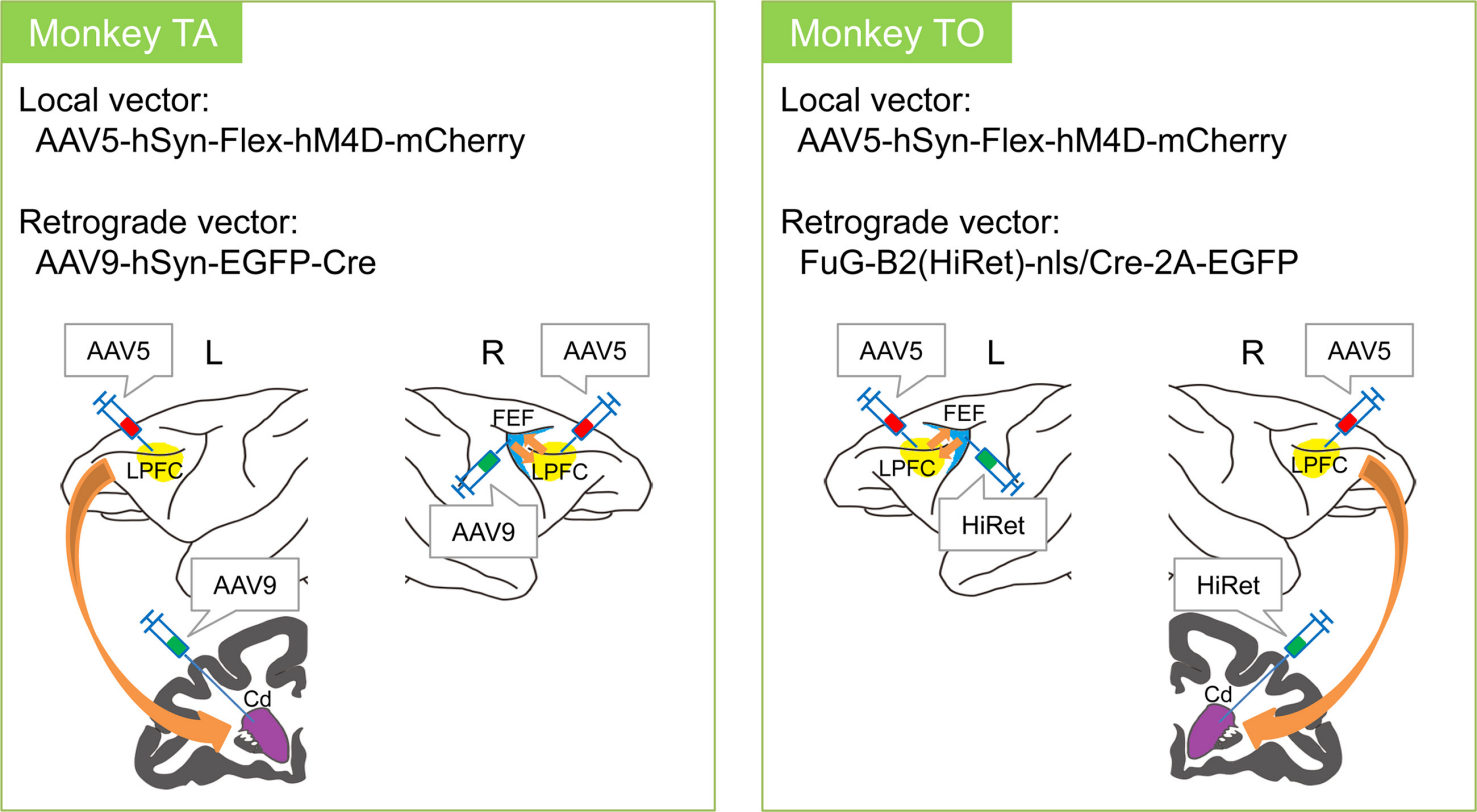
Target pathway and injected local and retrograde virus vectors. AAV5 was injected into the bilateral LPFC of both monkeys. AAV9 was injected into the left Cd and the right FEF of Monkey TA. HiRet was injected into the right Cd and the left FEF of Monkey TO.

The local vector was injected into the bilateral LPFC, and the retrograde vector was injected into either the Cd or the FEF for each hemisphere. AAV9 was injected into the left Cd and the right FEF of Monkey TA. HiRet was injected into the right Cd and the left FEF of Monkey TO (Fig 2). The LPFC is highly heterogeneous and contains various other projection neurons each of which connect with different brain areas. By using this double-infection technique, we can cause the expression of the target constructs only in the specific types of projection neurons in the LPFC (i.e., Cd-projection neurons and FEF-projection neurons).

### Injection protocol

In the macaque study, the monkeys were seated in a primate chair (with their heads fixed) inside a sound-attenuated and electrically shielded room in a P2A-level experimental area. Before virus vector injection, we monitored neural activities of the LPFC, the FEF, and the Cd with extra-cellular recording and identified the depth of neuron layers for each recording track. AC differential amplifier (MDA-4, BAK Electronics, FL, USA) was used to amplify neuronal action potentials. In each recording session, dura matter was first penetrated using a guide tube and then a tungsten electrode (FHC, ME, USA) was lowered using the NAN microdrive system (NAN instruments, Nazareth, Israel) through a recording grid (holes: 0.6 mm diameter and 1.0 mm apart from center to center; Nakazawa, Chiba, Japan). FEF sites were further confirmed by electrical microstimulation [28] as follows. After identifying the depth of the neuron layer of each FEF track, we repetitively delivered electrical stimulus (square wave, biphasic pulses, 0.20 ms positive and 0.20 ms negative pulse duration, frequency of 333 Hz, and 70 ms train duration) using an electrical stimulator with two isolation units (Nihon Koden, Tokyo, Japan) for every 1.0 mm of the neuron layer, with gaze monitored and recorded using an eye tracking system (ASL Eye-Trac 6000, MA, USA). We identified positions where evoked saccades were observed (< 50 μA) as FEF core regions. Injection positions were chosen only within neuron layers and, in the case of the FEF, within core regions that we identified. Two different depths per track in the LPFC were chosen as injection sites for AAV5, typically 0.5 mm and 1.5 mm above the bottom of the neuron layer. Two different depths per track in the FEF and three depths in the Cd were chosen as injection sites for AAV9/HiRet, typically 0.5 mm and 1.5 mm above the bottom of the neuron layer of the FEF and 2.0 mm, 4.0 mm, and 6.0 mm below the top of the neuron layer of the Cd. Following the identification of neuron layer and the determination of injection sites for each track, virus vector was injected using a microsyringe (Ito, Shizuoka, Japan) connected to a 25 G or 31 G needle. The needle was slowly lowered (0.2 mm/min) using the NAN microdrive system into the same track and stopped at 0.5 mm below the first injection site that was the deepest among target depths. After 1 min wait, the needle was withdrawn to the first injection site. The viral solution (2.0 μl) was then injected at a rate of 0.2 μl/min. The needle was maintained in place for extra 15 minutes for diffusion and then withdrawn to the second injection site. The same procedure was repeated for all remaining target depths.

We also conducted a mouse experiment to check the validity of the FLEX system. In this study, AAV5-hSyn-DIO-hM4D_i_-mCherry was injected unilaterally into the right cerebral cortex of the mouse under isoflurane anesthesia. The injection was conducted by a 10 micro litter syringe (Hamilton, NV, USA) connected to a 30 G needle for 20 min at the volume of 0.6 micro litter.

### Immunohistochemistry

One month after the last virus injection, the monkeys were deeply anesthetized with an intravenous injection of sodium pentobarbital (40 mg/kg, i.v.) and transcardially perfused with 0.01 M PBS and then 4% paraformaldehyde in 0.1 M phosphate buffer (pH 7.4). Brains were extracted and postfixed in 4% paraformaldehyde overnight at 4°C and cryoprotected with increasing gradients of sucrose (5, 10, 20%). Frozen brains were then sliced into coronal sections at a thickness of 30-μm using a cryostat.

One in four successive sections was immunohistochemically stained. Free-floating sections were washed in PBS and permeabilized in PBS containing 0.3% Triton X-100 (PBST). After blocked for 1 hour in 3% normal goat serum in PBST containing 1% bovine serum albumin (BSA-PBST), sections were incubated for 2 nights at 4°C with primary antibodies in BSA-PBST. eGFP was detected by a mouse anti-GFP antibody (1:250, Millipore, MA, USA), and mCherry was detected by a rabbit anti-RFP antibody (1:500, Abcam, Cambridge, UK). After washing in PBST, sections were incubated for 4 hours at room temperature with secondary antibodies in BSA-PBST. eGFP was visualized with Alexa-488 labeled goat anti-mouse IgG (1:1000, Molecular Probes, OR, USA), and mCherry was visualized with Alexa-568 labeled goat anti-rabbit IgG (1:1000, Molecular Probes, OR, USA). After washing in PBS, sections were mounted on glass slides with Fluoromount (Diagnostic BioSystems, CA, USA).

15 days after the AAV5 injection, the mouse brain was harvested under isoflurane anesthesia by 4% para-formaldehyde in 0.1 M PB at pH 7.4. The brains were removed, immersed in the same fixative overnight at 4°C, and then in 0.1 M PBS containing 15% sucrose at 4°C for 24 h. Then, these were immersed in 30% sucrose 0.1M PBS until they sunk. The brains were cut coronally at 25 μm on a freezing microtome. The immunostaining procedure was the same as that for mCherry procedure mentioned above.

### Fluorescence microscopy and 2D reconstruction

For the macaque brain, mCherry and eGFP fluorescence images were acquired using a camera lucida attached to an epifluorescence microscope (BX51, Olympus, Tokyo, Japan) with 10×, 20×, and 40× objective lenses. The number of mCherry-positive cells in LPFC slides (and eGFP-positive cells in some Cd/FEF slides) was counted manually with the epifluorescence microscope. Wide-area photomicrographs of the histological slices were taken under a light microscope (AZ100, Nikon, Tokyo, Japan) with a 1.25× objective lens. 2-dimensional (2D) reconstruction of injected brain areas was obtained by mapping mCherry- and eGFP-positive cells to corresponding wide-area photomicrographs using Photoshop and Illustrator (Adobe, CA, USA).

For the mouse brain, sections were captured using an Olympus digital camera attached to an epifluorescence microscope (BX52, Olympus, Tokyo, Japan). The needle tract was confirmed by tissue destruction under bright light microscopy.

## Results

### Local and double infection: LPFC-Cd

**Fig 3:**
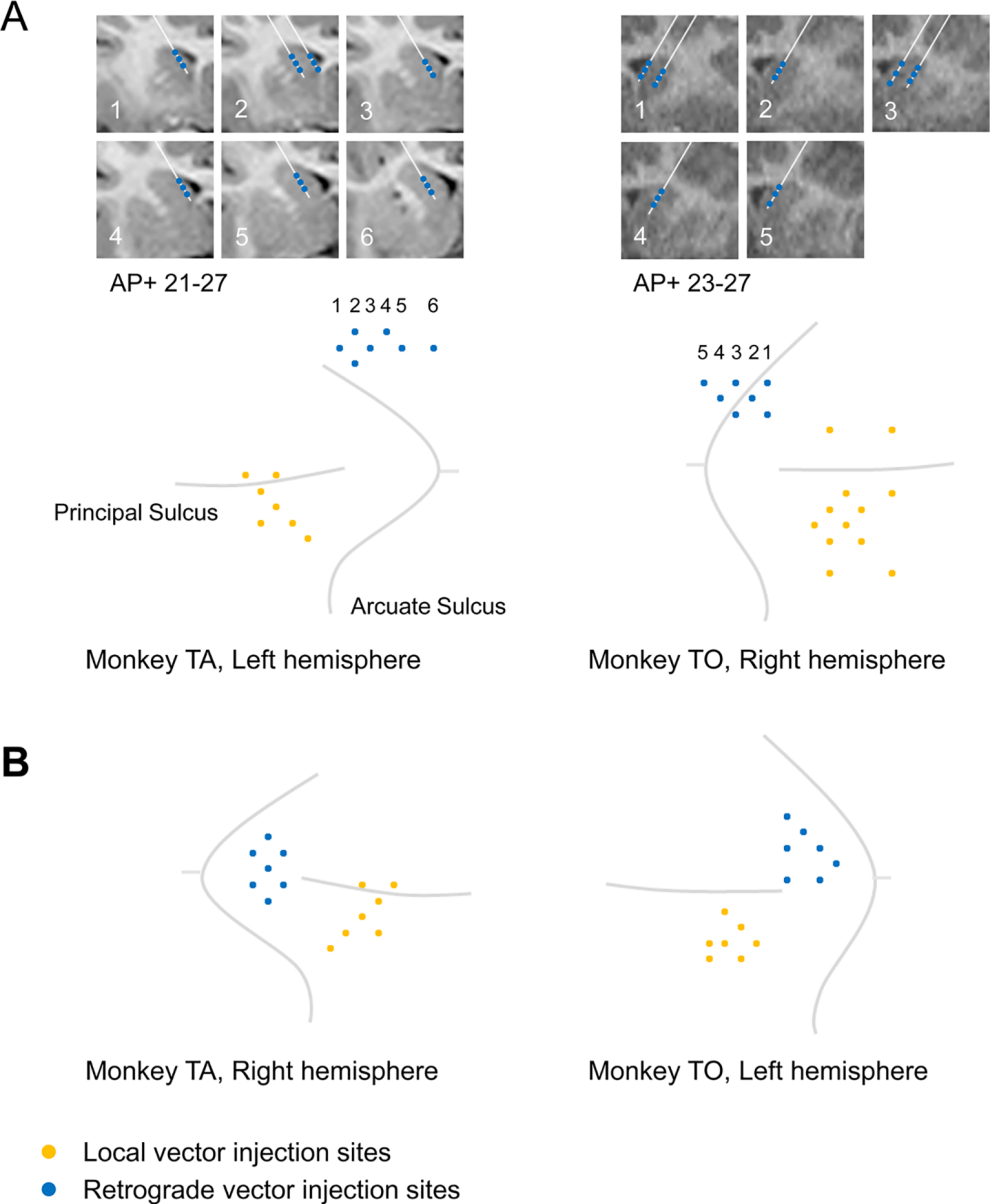
Anatomical location of insertion sites for the two monkeys. Distribution of insertion sites in the LPFC-Cd pathway (A), and in the LPFC-FEF pathway (B). The yellow circles represent insertion sites of local vector (AAV5 for the both monkeys). The blue circle represents insertion sites of retrograde vectors (AAV9 for Monkey TA; HiRet for Monkey TO). Three different depths along each injection track in the Cd were indicated by the blue circles on the T1 weighted anatomical images (A).

For Monkey TA (Fig 3A, left), we injected AAV9 along 7 tracks in the left Cd (the width of the injection area was 7 mm along the anterior-posterior (AP) direction) and AAV5 along 7 tracks in the ipsilateral LPFC (5 mm width along the AP direction). For Monkey TO (Fig 3A, right), we injected HiRet along 7 tracks in the right Cd (5 mm width along the AP direction) and AAV5 along 12 tracks in the ipsilateral LPFC (6 mm width along the AP direction).

**Fig 4:**
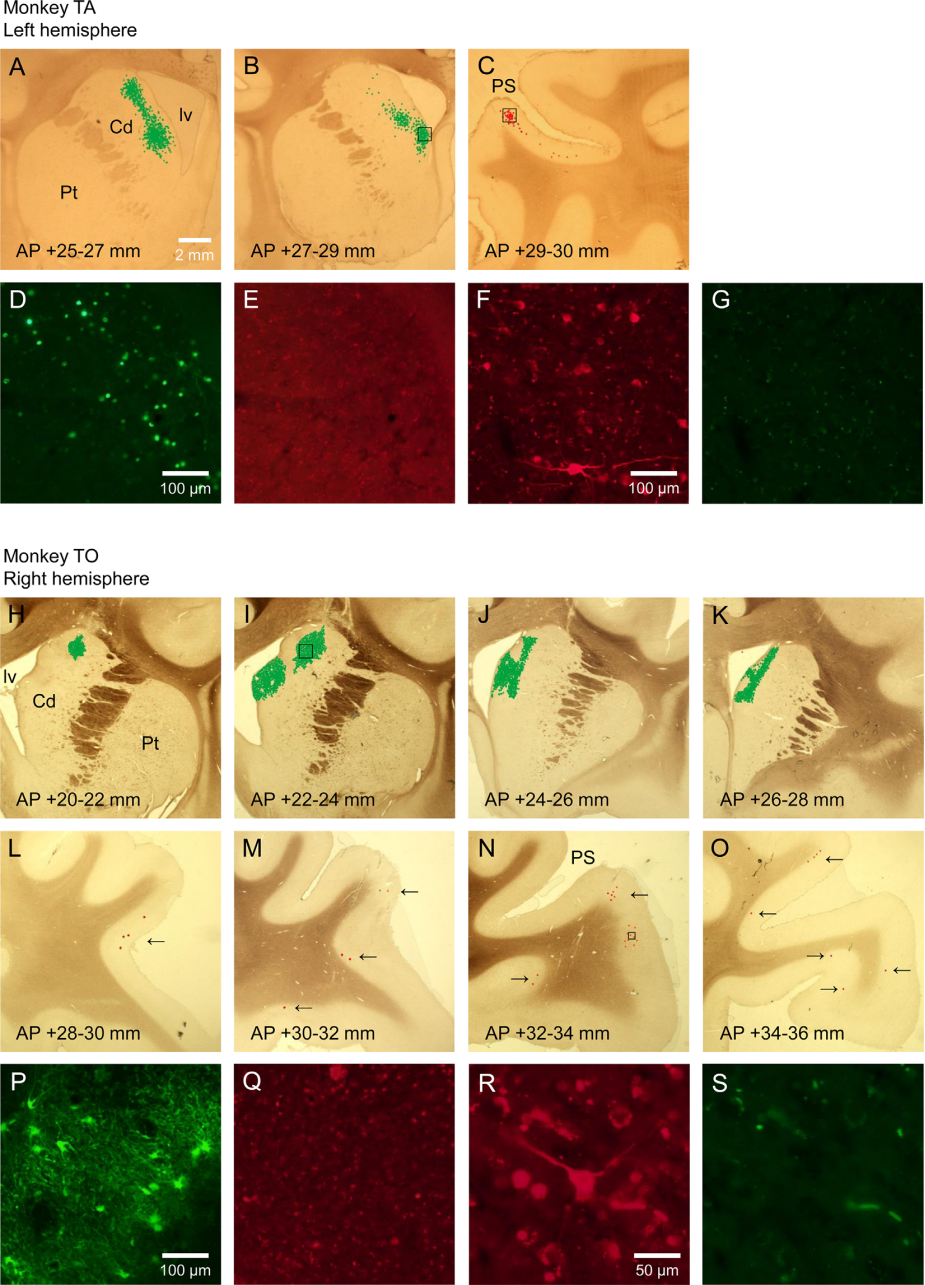
mCherry and eGFP expressions in the departure and the destination areas of the frontostriatal pathways. (A-C). Distribution of mCherry- and eGFP-positive cells in the left Cd and the ipsilateral LPFC of Monkey TA. Red dots represent mCherry-positive cells and green dots represent eGFP-positive cells. mCherry- and eGFP-positive cells were aggregated from several slides across ~2 mm along the AP direction and then superimposed on a corresponding wide-area photomicrograph. (D). eGFP-expressing cells in the boxed area of figure B as observed with a NIBA filter cube. (E). The micrograph of the same area as (D) as observed with a WIG filter cube. (F). mCherry-expressing cells in the boxed area of figure C as observed with a WIG filter cube. (G). The micrograph of the same area as (F) as observed with a NIBA filter cube. (H-O). Distribution of mCherry- and eGFP-positive cells in the right Cd and the ipsilateral LPFC of Monkey TO. Black arrows indicate where mCherry-expressing cells are. (P). eGFP-expressing cells in the boxed area of figure I as observed with a NIBA filter cube. (Q). The micrograph of the same area as (P) as observed with a WIG filter cube. (R). A mCherry-expressing cell in the boxed area of figure N as observed with a WIG filter cube. **(S)** The micrograph of the same area as (R) as observed with a NIBA filter cube. Cd: The caudate Nucleus, Pt: The putamen, lv: The lateral ventricle, PS: The principal sulcus.

Locally infected eGFP-positive cells were observed near the lateral ventricle in the left Cd of Monkey TA across 4.3mm along the AP direction (Fig 4A, 4B, 4D and 4E). These cells were infected from their cell bodies and/or from their axon terminals in the injection sites. eGFP-positive cells were highly frequent in the Cd head (over 100 cells in several corresponding slides) and gradually decreased with distance from the Cd head along the AP direction. We could not find eGFP-positive cells in the posterior part of Cd injection sites. In the left LPFC of the same monkey, we observed double-infected mCherry-positive cells in the inferior bank of the principal sulcus across 1.0 mm along the AP direction (in total, 81 cells: Fig 4C, 4F and 4G). We found relatively large number of mCherry-positive cells (50 cells) in the slide that is around the most posterior AAV5 injection site. We could not find mCherry-positive cells in the anterior part of LPFC injection sites. Theoretically, mCherry-positive cells also could be eGFP-positive, but all mCherry-positive cells we found were eGFP-negative, presumably because the amount of eGFP expression was insufficient for fluorescence observation in these cells.

High frequency of infected eGFP-positive cells were observed near the dorsal part of the lateral ventricle in the right Cd of Monkey TO across 6.7mm along the AP direction (Fig 4H, 4I, 4J, 4K, 4P and 4Q). These cells were presumably medium spiny neurons infected from their collateral axon terminals and/or interneurons infected from their nearby axon terminals. In contrast to the observation of AAV9 injection sites, dense neural fibers were observed around these eGFP-positive cell bodies since HiRet contained a self-cleaving 2A peptide between Cre and eGFP. In the ipsilateral LPFC, we observed low frequency of mCherry-positive cells in the superior bank of the principal sulcus and the ventrolateral prefrontal cortex (VLPFC) across 7.2mm along the AP direction (in total, 30 cells: Fig 4L, 4M, 4N, 4O, 4R and 4S). We also found 5 mCherry-positive cells in the right orbitofrontal cortex (OFC), which might be caused by the leak of vector solution through a thin white matter layer between LPFC and OFC. All mCherry-positive cells we found are eGFP-negative.

In the both hemispheres, no eGFP-positive cells were observed around track traces in the cortex through which AAV9/HiRet had been injected into the Cd (S1 Fig), indicating that infection by these retrograde vectors was confined within the Cd and that no leak of vector solution occurred.

### Local and double infection: LPFC-FEF

For Monkey TA (Fig 3B, left), we injected AAV9 along 7 tracks in the right FEF (3 mm width along the AP direction) and AAV5 along 7 tracks in the ipsilateral LPFC (5 mm width along the AP direction). For Monkey TO (Fig 3B, right), we injected HiRet along 7 tracks in the left FEF (4 mm width along the AP direction) and AAV5 along 7 tracks in the ipsilateral LPFC (4 mm width along the AP direction).

**Fig 5:**
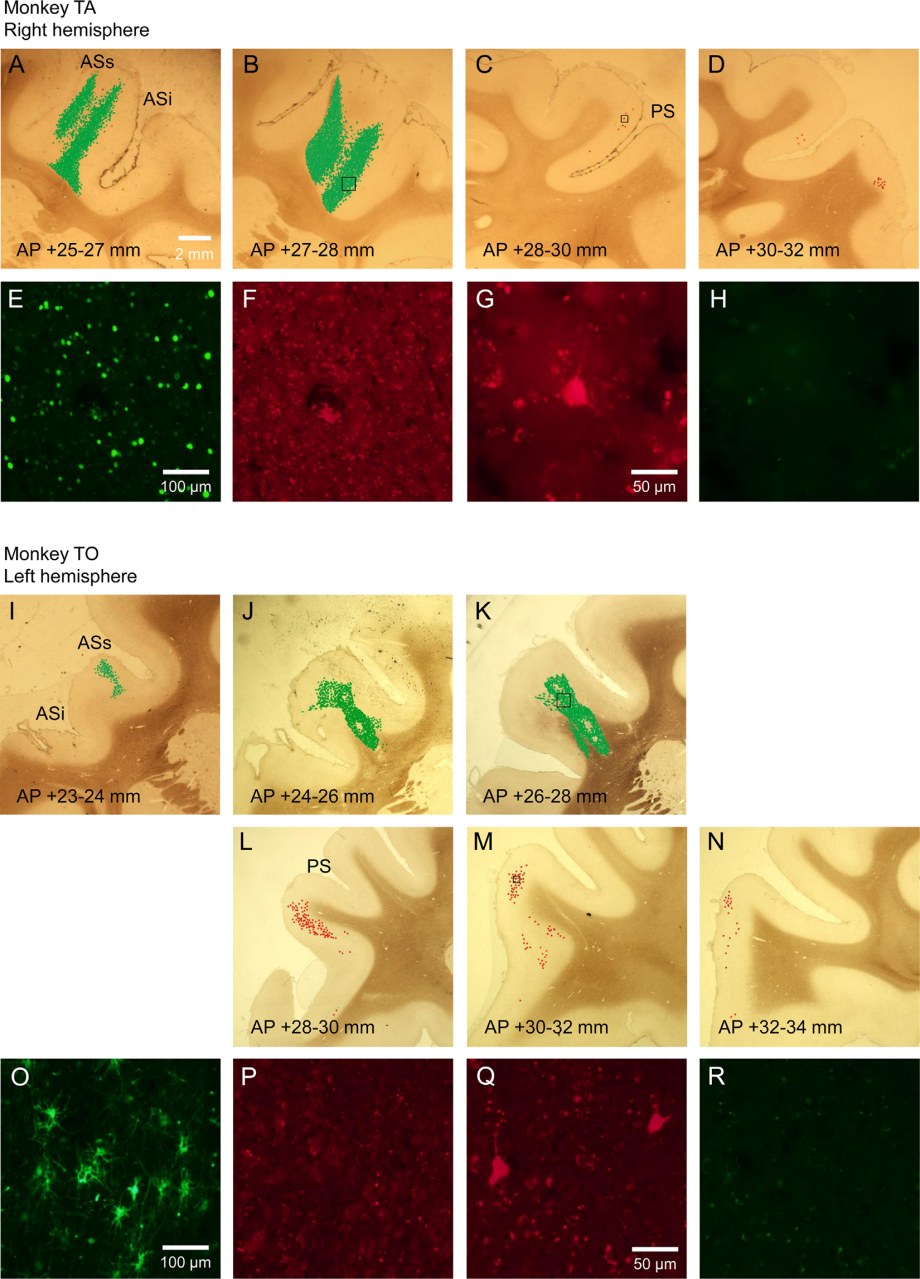
mCherry and eGFP expressions in the departure and the destination areas of the frontofrontal pathways. (A-D). Distribution of mCherry- and eGFP-positive cells in the right FEF and the ipsilateral LPFC of Monkey TA. (E). eGFP-expressing cells in the boxed area of figure B as observed with a NIBA filter cube. (F) The micrograph of the same area as (E) as observed with a WIG filter cube. (G). A mCherry-expressing cell in the boxed area of figure C as observed with a WIG filter cube. (H) The micrograph of the same area as (G) as observed with a NIBA filter cube. (I-N). Distribution of mCherry- and eGFP-positive cells in the left FEF and the ipsilateral LPFC of Monkey TO. (O). eGFP-expressing cells in the boxed area of figure K as observed with a NIBA filter cube. (P) The micrograph of the same area as (O) as observed with a WIG filter cube. (Q). mCherry-expressing cells in the boxed area of figure M as observed with a NIBA filter cube. (R) The micrograph of the same area as (Q) as observed with a NIBA filter cube. ASs: The superior ramus of the arcuate sulcus, ASi: The inferior ramus of the arcuate sulcus, PS: The principal sulcus.

Locally infected eGFP-positive cells were observed along several track traces in the right FEF of Moneky TA across 4.8 mm along the AP direction (Fig 5A, 5B, 5E and 5F). The frequency of eGFP expression in the area was higher (over 1,000 cells in several corresponding slides) than that in the left Cd of the same monkey. In the ipsilateral LPFC, we observed a low frequency of double-infected mCherry-positive cells in the superior bank of the principal sulcus and the VLPFC across 3.4 mm along the AP direction (in total, 24 cells: Fig 5C, 5D, 5G and 5H). All mCherry-positive cells we found were eGFP-negative.

A high frequency of eGFP-positive cells were observed in the left FEF of Monkey TO across 4.8 mm along the AP direction (Fig 5I, 5J, 5K, 5O and 5P). eGFP was densely expressed also in the fibers of these cells as in the right Cd of the same monkey. In the ipsilateral LPFC, mCherry-positive cells were observed from the entrance of the lower side of the principal sulcus to the VLPFC across 5.3 mm along the AP direction (in total, 221 cells: Fig 5L, 5M, 5N, 5Q and 5R). The expression of these mCherry-positive cells was more frequent than that in the right LPFC of Monkey TA. All mCherry-positive cells we found were eGFP-negative.

### Single local infection to the mouse cortex

In the mouse control experiment, the Cre-inducible AAV5 virus was injected into the cerebral cortex without Cre-virus in order to exclude the possibility of an autonomous viral DNA mutation event that caused the Cre-independent expression of mCherry. As seen in S2 Fig, mCherry was not detectable without Cre-virus. This potential confound, therefore, can be ruled out.

## Discussion

Artificially modulating neural activities of specific neurons using the double virus vector infection is a potential method to uncover functions of specific neural circuits when being combined with optogenetic and pharmacogenetic technologies. This method should be especially useful for neurophysiological studies using macaque monkeys because germline modification (a tool that is commonly used in rodent studies and recently begin to be applied to marmoset studies) is unavailable for them. Here, we examined the feasibility of the double virus vector infection method in the frontostriatal (LPFC-Cd) and the frontofrontal (LPFC-FEF) pathways in the macaque brain. To achieve selective transfection of neurons that project along a specific neural pathway, the double infection system utilizes two different vectors for the departure and the destination areas of the pathway. We chose AAV5 as a locally infectable vector that was injected into the LPFC and AAV9/HiRet as retrogradely infectable vectors that were injected into the Cd/FEF. Doubly-transfected neurons, labeled by mCherry expression, were found in the LPFC in all of the four injected hemispheres, suggesting that the double virus vector infection is workable for neural pathways in the primate prefrontal network, selective modification of which is indispensable for uncovering neural substrates of higher cognitive functions.

One possible concern about our results is that the mCherry-positive cells we observed might be derived either from Cre-independent mCherry expression caused by autonomous viral DNA mutation or from extracellular diffusion of AAV9/HiRet into AAV5-infected cells instead of intracellular retrograde axonal transport. The former possibility was ruled out by our mouse study. We injected AAV5 alone into its cerebral cortex and found no mCherry-positive cells. In addition to this, we did not find any mCherry-positive cells at several AAV5-injected tracks in the macaque brain, for instance, at the anterior part of the left LPFC of Monkey TA. These observations indicate that the FLEX system we used was not able to function without Cre and thus effectively prevented leaky mCherry expression. Likewise, the latter possibility was ruled out by several observations. In the two hemispheres where retrograde vectors were injected into the Cd, no eGFP expression was found around the track traces outside the Cd, indicating that leak of vector solution along injection cannulas did not occur. While the distance from the Cd to the LPFC is long enough to prevent direct passive diffusion of the vector solution, that from the FEF to the LPFC is not. We thus carefully choose injection sites in the FEF and the LPFC in such a way that the distance between the nearest injection sites was more than 4 mm. Furthermore, mCherry-positive cells were broadly distributed around injection sites in the LPFC ipsilateral to the injected FEF. This distribution pattern cannot be explained solely by passive diffusion of the vector solution. Therefore, the concern that the mCherry expression we found was derived from some route other than double virus vector infection can be excluded.

Although all injected LPFC areas were shown to contain doubly-infected mCherry-positive neurons, the number of these doubly-infected neurons was not so large. There are several possible reasons behind this sparseness of doubly-infected neurons. We focus on two among them. First, we could not cover entire regions of the Cd/FEF by injected retrograde vectors. Neural connections from the injected LPFC regions to the covered Cd/FEF regions might be poorer than that from the same LPFC regions to the non-covered Cd/FEF regions. In this study, mCherry-positive neurons were mainly found in the ventral side of the principal sulcus (VLPFC). Previous studies suggested that the most dense projection area from the VLPFC neurons lies in the lateral part of Cd while that from dorsolateral PFC (DLPFC) neurons lies in the more medial part [12, 13, 14]. In both monkeys of this study, we found eGFP-positive neurons which labeled actual injection sites of the retrograde vectors, in the medial part of the Cd. While axon terminals of the LPFC projection neurons were widely spread across the Cd, we might have missed the core terminal regions from the injected source areas. Likewise, a recent study suggested that the caudal part of the VLPFC (area 45B) most frequently projects to the middle part of the FEF, while the rostral part of the VLPFC (area 45A) projects to the more dorsal part of the FEF [15]. We found mCherry-positive neurons mainly in 45A and eGFP-positive neurons around the middle part of the FEF, a bit ventral to the suggested core area projected from 45A. Therefore, more extensive injection of retrograde vectors (sufficient enough to cover remaining destination areas) may be required to obtain a larger number of doubly-infected neurons.

Second, the efficacy of retrograde vectors used in this study might be not enough to achieve high retrograde gene transfer along the pathways we targeted in the macaque brain. Previous studies using AAV showed that the directionality and the efficacy of its gene transfer are dependent on several factors, such as serotype, animal species, and target pathway [18, 19, 29]. The retrograde gene transfer of AAV9 has been shown in different pathways of the rat and the marmoset brains [20, 30, 31]. In this study, we demonstrated retrograde gene transfer of AAV9 from both cortical and subcortical brain areas in the macaque brain. The efficacy of this transfer, however, has not been compared with that of different AAV serotypes. AAV9 thus has not yet been confirmed as the best choice for retrograde axonal transport along our target pathways among other serotypes, for instance, AAV6 [32]. Although it is difficult, due to limited sample size, to compare the efficacy of retrograde gene transfer of AAV9 with that of HiRet from our result, we observed similar sparsely-spread patterns of mCherry-positive neurons in the two LPFC ipsilateral to injected FEF areas and the number of doubly-infected neurons in the left LPFC of the HiRet injected monkey was approximately ten times larger than that in the right LPFC of the AAV9 injected monkey. This result, together with other studies testing the efficacy of modified lentivirus based retrograde vectors [23, 33], suggests that HiRet is a prospective candidate for bringing high retrograde gene transfer in the higher primate brain among other adeno-associated virus and lentivirus based vectors. Still, there is plenty of room left for improving its efficacy of axonal transport [34]. Further studies, therefore, should be done with improved retrograde vectors to achieve more frequent double-infection.

We injected AAV5 harboring hM4D_i_, the inhibitory type of the newly-developed designer receptors called DREADDs, within the “Cre-On” FLEX double-floxed sequence. The reason for this inclusion was to establish the feasibility of DREADD expression in specific prefrontal neurons of the macaque brain. Activities of neurons that express DREADDs can be modulated by administering the extrinsic ligand CNO [24, 25]. With this pharmacogenetic tool, manipulations of specific cell-types and resultant behavioral changes have been demonstrated in rodents and *Drosophila* [6, 35, 36, 37, 38]. Applying this tool in combination with the double infection technique into the primate brain will shed light on how our brain drives higher cognitive functions and how its disturbance results in various psychiatric disorders. When applying to primates, the DREADD system might be advantageous than optogenetics at least in the following two respects. First, pharmacogenetic method can cover large volumes of tissues since neural modulation of DREADD-expressing cells can be evoked by the systemic administration of CNO. The result of a recent study comparing optogenetic and electrical perturbations suggested that optogenetics has difficulty in bringing about appropriate behavioral changes in macaques since neural modulation of spiking activities across a significant broad area is required [39]. CNO administration may evoke neural modulation in a large number of cells enough to bring behavioral changes as long as the adequate expression of DREADDs in these cells has been achieved by, for instance, extensive double virus vector injection. Second, the pharmacogenetic method is less invasive than optogenetics since the former does not require intracranial placement of stimulating devices such as optical fibers. CNO exclusively affects DREADDs and has been proven not to be cytotoxic [40]. Various psychiatric disorders are suggested to be related with hyper- or hypoactivity of specific neural pathways such as the frontostriatal pathway [9, 10, 11]. Pathway-specific neural regulation using the DREADD system, therefore, has a bigger potential to lead to clinical application. We hope that this study provides a step toward such a direction.

## Supporting Information

S1 Fig eGFP expression around the track traces. (A) The cortex of the left hemisphere of Monkey TA around a Cd-injection trace. Immunohistochemical response to eGFP was negative. (B) The same area as (A) as observed with a WIB filter cube. (C) The cortex of the right hemisphere of Monkey TO around a Cd-injection trace. Immunohistochemical response to eGFP was negative. (D) The same area as (C) as observed with a WIB filter cube.
(TIF)

S2 Fig mCherry expression in the mouse brain. (A) Cre-inducible AAV5 virus was injected into the cerebral cortex of the mouse without Cre-virus. Immunohistochemical response to mCherry was negative.
(TIF)
