# Supplementary figures and images for: Double Virus Vector Infection to the Prefrontal Network of the Macaque Brain
Source: PLoS One. 2015 Jul 20;10(7):e0132825. doi: 10.1371/journal.pone.0132825 (PMC4507872; doi:10.1371/journal.pone.0132825)

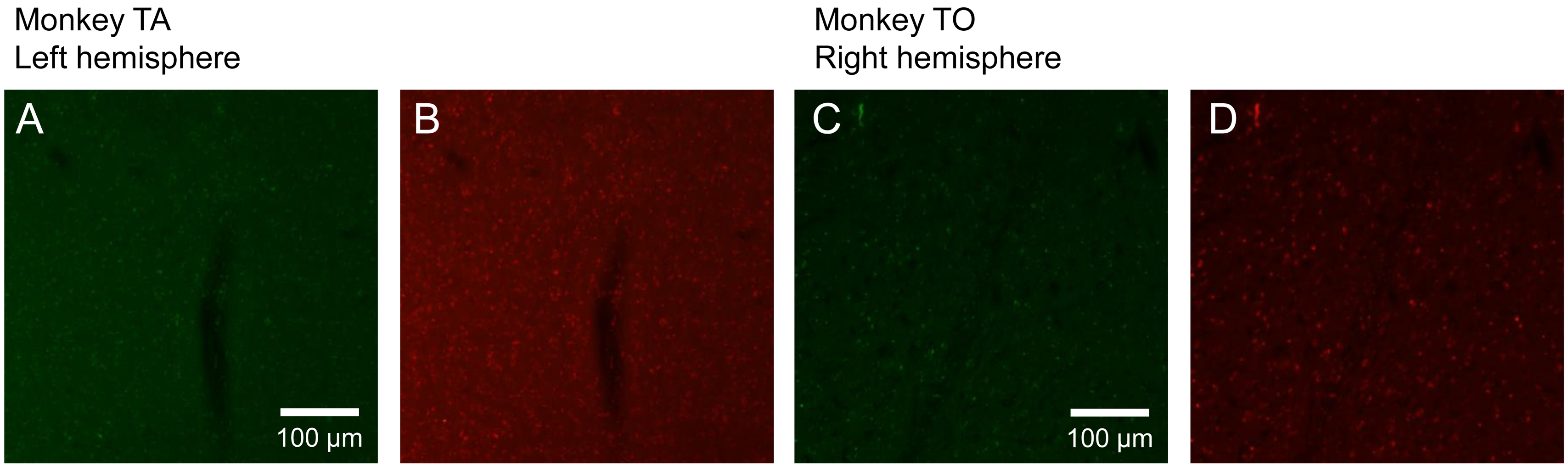

Supplement: S1 Fig — (A) The cortex of the left hemisphere of Monkey TA around a Cd-injection trace. Immunohistochemical response to eGFP was negative. (B) The same area as (A) as observed with a WIB filter cube. (C) The cortex of the right hemisphere of Monkey TO around a Cd-injection trace. Immunohistochemical response to eGFP was negative. (D) The same area as (C) as observed with a WIB filter cube. (TIF) [file pone.0132825.s001.tif]

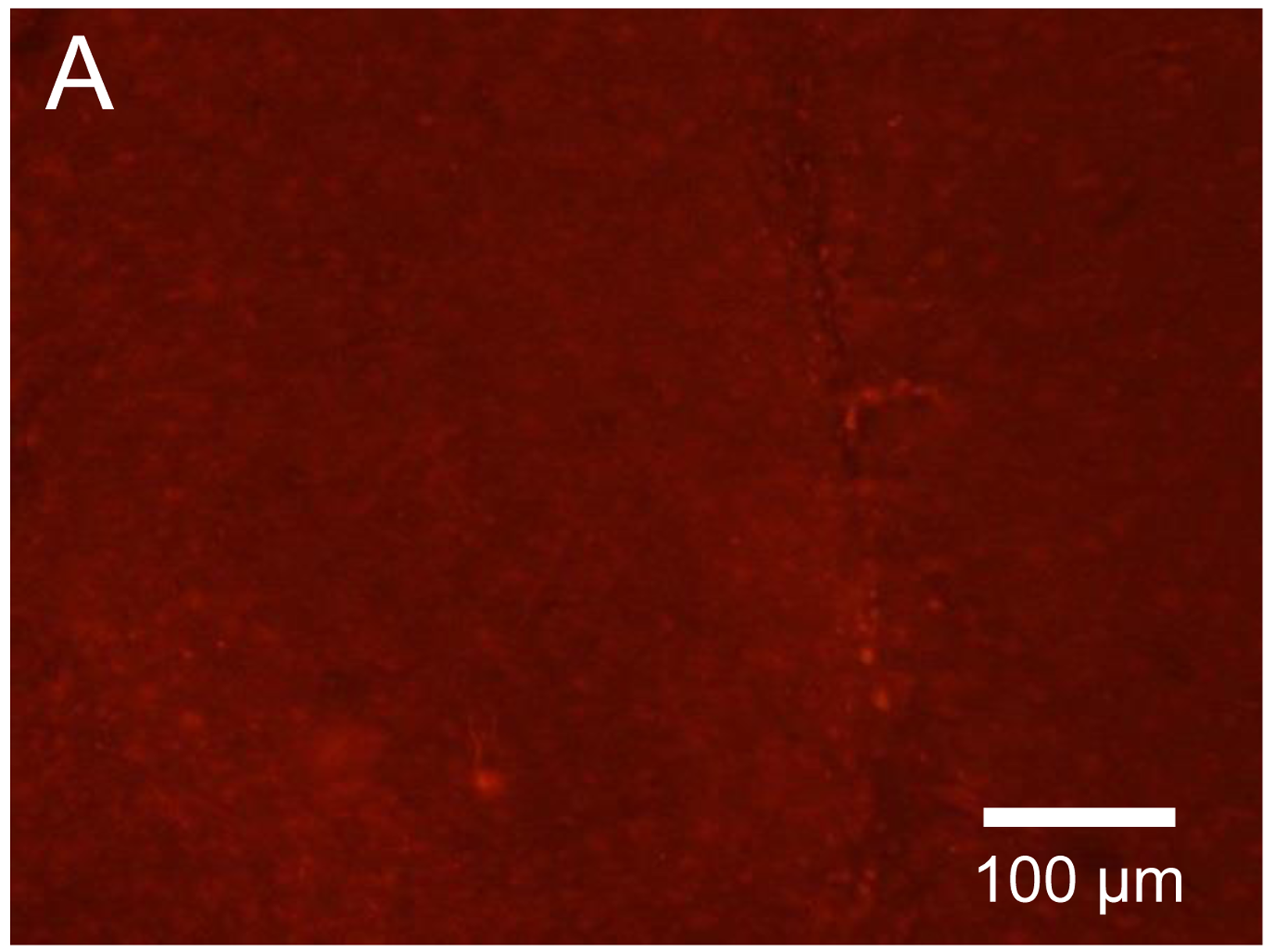

Supplement: S2 Fig — (A) Cre-inducible AAV5 virus was injected into the cerebral cortex of the mouse without Cre-virus. Immunohistochemical response to mCherry was negative. (TIF) [file pone.0132825.s002.tif]
